# Novel cis-Pt(II) Complexes with Alkylpyrazole Ligands: Synthesis, Characterization, and Unusual Mode of Anticancer Action

**DOI:** 10.1155/2022/1717200

**Published:** 2022-03-02

**Authors:** Jana Kasparkova, Hana Kostrhunova, Vojtech Novohradsky, Аlexey A. Logvinov, Viktor V. Temnov, Nataliya E. Borisova, Tatiana A. Podrugina, Lenka Markova, Pavel Starha, Alexey. A. Nazarov, Viktor Brabec

**Affiliations:** ^1^Czech Academy of Sciences, Institute of Biophysics, Brno, CZ-61265, Czech Republic; ^2^Lomonosov Moscow State University, Faculty of Chemistry, Leninskie Gory 1/3, Moscow 119991, Russia

## Abstract

One concept of improving anticancer effects of conventional platinum-based antitumor drugs consists of conjugating these compounds with other biologically (antitumor) active agents, acting by a different mechanism. Here, we present synthesis, physicochemical characterization, biological effects, and mechanisms of action of four new analogs of conventional cisplatin, namely, cis-Pt(II) complexes containing either methyl or ethyl pyrazole N-donor ligands and chlorido or iodido ligands. It is noteworthy that while chlorido complexes display activity in a variety of cancer cell lines comparable to cisplatin, iodido complexes are considerably more potent due to their enhanced hydrophobicity and consequently enhanced cellular accumulation. Moreover, all of the studied Pt(II) alkylpyrazole complexes display a higher selectivity for tumor cells and effectively overcome the acquired resistance to cisplatin. Further results focused on the mechanism of action of the studied complexes and showed that in contrast to cisplatin and several platinum-based antitumor drugs, DNA damage by the investigated Pt(II)-alkylpyrazole complexes does not play a major role in their mechanism of action. Our findings demonstrate that inhibition of the tubulin kinesin Eg5, which is essential for forming a functional mitotic spindle, plays an important role in their mechanism of antiproliferative action.

## 1. Introduction

Cisplatin, carboplatin, and oxaliplatin are the only worldwide approved metal-based antitumor drugs [1–4]. For decades, the search for new antitumor Pt drugs followed the rules established based on knowledge of the mechanism of action of cisplatin and its direct analogs. However, this search is now shifting to the greater structural diversity of platinum complexes, including nonclassical structures [5–16]. Cis-Pt(II) iodido complexes have long been predominantly used as intermediates in synthesizing target platinum chlorides and carboxylates, particularly by the Dhara method or its modifications [17, 18]. However, later several studies have shown that the iodido ligand may have unique chemical, physical, and biological properties, with a mechanism of action different from that of Pt(II) chlorido analogs [19–22]. For example, cis-Pt(II) diiodides containing 7-azaindole and its derivatives were significantly more active than cisplatin in several cancer cell lines [19].

The use of heterocyclic ligands instead of simple amines makes it possible to tune the activity of Pt complexes. In addition, the large surface area allows more efficient and stronger interactions with DNA bases by *π*-*π*-stacking or altered interactions with DNA; also, the presence of a hindered group can prevent the inactivation of complexes by reactions with sulfur-containing ligands [22–26]. Pioneering work on the synthesis of Pt(II) complexes by Jan Reedijk and his coworkers in the 1970s provided 1H-imidazole, 1-methyl-1H-imidazole, and 1H-pyrazole complexes, which were investigated by a wide range of physicochemical methods [27, 28]. Later, asymmetric cis-Pt(II) complexes with isopropylamine, 1-methyl-1H-pyrazole, or 1-methyl-1H-imidazole ligands were also synthesized, and their *in vitro* antiproliferative activity and interactions with DNA bases were investigated by the Navarro–Ranninger group [29]. This article reports the preparation and physicochemical characterization of new cis-Pt(II) complexes with 1-alkyl-1H-pyrazole ligands containing the iodido or chlorido leaving groups. We also turned our attention to investigating antiproliferative activity in cancer cells and some unique aspects of the mechanism of action in further pursuit of novel platinum cancer drug candidates.

## 2. Materials and Methods

### 2.1. The Chemicals

The purchased reagents were as follows: K_2_[PtCl_4_] (Fluorochem), 1*H*-pyrazole (Sigma-Aldrich), and 3-methyl-1*H*-pyrazole (mepz) (Sigma-Aldrich). All solvents were purified and degassed before use. ^1^H NMR, ^13^C NMR, and ^195^Pt NMR spectroscopy were performed at 298 K on either Bruker AM-400 or Bruker Avance 600. ^1^H and ^13^C NMR spectra were calibrated against the residual solvent of DMF-*d*_*7*_ (^1^H NMR (8.03, 2.92 and 2.75 ppm) and ^13^C NMR (163.2, 34.9, and 29.8 ppm). ^195^Pt NMR spectra were calibrated by external reference (H_2_[PtCl_6_] in D_2_O, *δ* = 0 ppm). The splitting of proton resonances in the reported ^1^H NMR spectra is defined as *s* = singlet, *d* = doublet, *t* = triplet, *q* = quadruplet, br = broad band, and *m* = multiplet. High-resolution electrospray ionization mass spectrometry (ESI-MS) was performed by Shimadzu IT-TOF ion trap spectrometer in positive (ESI+) ionization modes of the DMF solutions.

### 2.2. General Procedure for the Synthesis of Pt Diiodido Complexes

K_2_[PtCl_4_] (0.5 mmol) was dissolved in 10 mL of deionized water at room temperature, and the 24 equivalents of KI (12 mmol) were added to the red solution, which turned dark brown over 30 min of stirring in the dark. Then, desired pyrazole was added in one portion, and the reaction mixture was stirred overnight at room temperature. The formed brown solid was removed by filtration and washed with deionized water (3 × 5 mL), methanol (3 × 2 mL), and diethyl ether (3 × 5 mL). The products were dried under a vacuum.

*cis*-[Pt(*mepz*)_2_I_2_] (**1a**): ^1^H NMR (600 MHz, DMF-*d*_7_, 298 K): *δ* 4.43 (s, 6H, CH_3_); 6.48 (m, 2H, C^4^H); 8.10 (m, 4H, C^3^H, C^5^H) ppm. ^13^C NMR (125 MHz, DMF-*d*_7_, 298 K): *δ* 41.12, 108.28, 136.04, 142.36 ppm. ^195^Pt NMR (128 MHz, DMF-*d*_*7*_, 298 K): *δ* −3247 ppm. MS (ESI) *m/z* 613.8879 [M + H]^+^, 635.8697 [M + Na]^+^, 485.9755 [M − I]^+^. Yield = 250 mg (83%).

*cis*-[Pt(*etpz*)_2_I_2_] (**1b**): ^1^H NMR (600 MHz, DMF-*d*_7_, 298 K): *δ* 1.58 (t, 6H, *J* = 7.3 Hz, CH_3_), 4.96–5.09 (m, 4H, CH_2_), 6.53 (m, 2H, C^4^H); 8.11 (d, 2H, *J* = 1.9 Hz, C^3^H); 7.97 (d, 2H, *J* = 2.5 Hz, C^5^H) ppm. ^13^C NMR (125 MHz, DMF-*d*_7_, 298 K): *δ* 15.65, 48.96, 108.81, 133.89, 142.16 ppm. ^195^Pt NMR (128 MHz, DMF-*d*_7_, 298 K): *δ* −3243 ppm. MS (ESI) *m/z* 641.9194 [M + H]^+^, 663.8983 [M + Na]^+^, 514.0043 [M − I]^+^. Yield = 202 mg (65%).

### 2.3. General Procedure for the Synthesis of Pt Dichlorido Complexes

Suspension of **1a** or **1b** (0.5 mmol) in 5 mL of deionized water was treated with AgNO_3_ (0.95 mmol), and the reaction mixture was stirred overnight at room temperature in the dark. The precipitate of silver iodide was filtered off, and 20 equivalents of KCl (10 mmol) were added and stirred for 24 h in the dark. The obtained yellow solid was removed by filtration and washed with deionized water (3 × 3 mL), acetone (3 × 1 mL), and diethyl ether (3 × 5 mL). The products were dried under a vacuum.

*cis*-[Pt(*mepz*)_2_Cl_2_] (**2a**): ^1^H NMR (600 MHz, DMF-*d*_7_, 298 K): *δ* 4.40 (s, 6H, CH_3_); 6.45 (m, 2H, C^4^H); 8.04 (d, *J* = 2.4 Hz, 2H, C^3^H); 8.12 (d, *J* = 2.4 Hz, 2H, C^5^H) ppm. ^13^C NMR (150 MHz, DMF-*d*_7_, 298 K): *δ* 40.33, 108.22, 135.97, 142.99. ^195^Pt NMR (128 MHz, DMF-*d*_7_, 298 K): *δ* −2019 ppm. MS (ESI) *m/z* 431.0150 [M + H]^+^, 452.9965 [M + Na]^+^, 394.0388 [M − Cl]^+^. Yield = 142 mg (66%).

*cis*-[Pt(*etpz*)_2_Cl_2_] (**2b**): ^1^H NMR (600 MHz, DMF-*d*_*7*_, 298 K): *δ* 1.59 (*t*, *J* = 7.3 Hz, 6H, CH_3_); 5.00 (q, *J* = 7.3 Hz, 4H, CH_2_); 6.50 (m, 2H, C^4^H); 8.02 (d, *J* = 2.2 Hz, 2H, C^3^H); 8.18 (d, *J* = 2.5 Hz, 1H, C^5^H) ppm. ^13^C NMR (150 MHz, DMF-*d*_7_, 298 K): *δ* 15.63, 48.61, 108.76, 133.97, 142.68 ppm. ^195^Pt NMR (128 MHz, DMF-*d*_7_, 298 K): *δ* −2004 ppm. MS (ESI) *m/z* 459.0456 [M + H]^+^, 481.0279 [M + Na]^+^, 423.0691 [M − Cl]^+^. Yield = 121 mg (53%).

### 2.4. ^1^H NMR Stability Studies

An appropriate amount of the complexes **1a**,**b** and **2a**,**b** was dissolved in 200 *µ*L of DMF-*d*_7_ and 300 *µ*L of D_2_O was added (1 mM final concentration). ^1^H NMR spectra (Varian spectrometer (Varian Inc.; Palo Alto, CA, USA), 400 MHz, 298 K) of these solutions were recorded at various time points (0–48 h), and the solutions were stored at r.t. in the dark between the individual experiments. The spectra were referenced to the residual signal of water (4.79 ppm). *Note*: DMF-*d*_7_ ensured the solubility of the complexes, as their solubility in water is not sufficient for ^1^H NMR. Deuterated solvents DMF-*d*_7_ and D_2_O were supplied by Merck/Sigma-Aldrich (Prague, Czech Republic). Free pyrazoles were studied by ^1^H NMR under the same experimental conditions (for comparative purposes).

### 2.5. Material and Cell Lines for Biological Studies

Cisplatin, N,N-dimethylformamide (DMF), and dimethylsulfoxide (DMSO) were from Sigma-Aldrich (Prague, Czech Republic). MTT was from Calbiochem (Darmstadt, Germany). Stock solutions for cellular studies were prepared by dissolving Pt compounds in DMF to a final concentration of 5 or 20 mM and serially diluted before testing in cell culture media. To avoid DMF toxicity, the final DMF concentration in the cell culture medium did not exceed 0.5% v/v.

The human ovarian carcinoma cisplatin-sensitive A2780 cells, cisplatin-resistant A2780cisR, and human colorectal carcinoma cells HCT116 were kindly supplied by Professor B. Keppler, University of Vienna (Austria). Highly invasive breast carcinoma MDA-MB-231 cells, human rhabdomyosarcoma (RD) cells, and human MRC-5 pd30 cells derived from normal lung tissue were purchased from the European Collection of Authenticated Cell Cultures (ECACC; Salisbury, UK). Chinese hamster ovary CHO-K1 cell line (wild-type) and its derivative MMC-2 carrying the ERCC3/XPB mutation (NER-deficient) cell line were kindly supplied by Dr. M. Pirsel, Cancer Research Institute, Slovak Academy of Sciences, Bratislava (Slovakia). The A2780, A2780cisR, and RD cells were grown in RPMI 1640 medium (Biosera, Boussens, France) supplemented with gentamycin (50 mg/mL, Serva, Heidelberg, Germany) and 10% heat-inactivated fetal bovine serum (PAA, Pasching, Austria). The acquired resistance of A2780cisR cells was maintained by supplementing the medium with 1 *µ*M cisplatin repeatedly every second passage. The HCT116, MDA-MB-231, MRC5 pd30, CHO-K1, and MMC-2 cells were grown in DMEM medium (Dulbecco's Modified Eagle's Medium, high glucose (4.5 gL^−1^, PAA) supplemented with gentamycin (50 mgmL^−1^, Serva) and 10% heat-inactivated fetal bovine serum (PAA). The cells were cultured in an incubator at 37°C in a humidified 5% CO_2_ atmosphere and subcultured 2–3 times a week.

### 2.6. Antiproliferative Activity

Effects of the platinum complexes on cell proliferation were evaluated using commonly used MTT [3-(4,5-dimethyl-2-thiazolyl)-2,5-diphenyl-2H-tetrazolium bromide] as already described [30]. Briefly, the cells were seeded in 96-well tissue culture plates at a density of 1 × 10^4^ A2780/cisR cells/well, 3 × 10^3^ MDA-MB-231, RD, CHO-K1, or MMC-2 cells/well, 1 × 10^4^ MRC-5 pd30 cells/well, 1.5 × 10^3^ HCT-116 cells/well, in 100 *μ*L of medium and kept overnight at 37 °C in a 5% CO_2_ humidified atmosphere. Then, the cells were treated with the tested compounds in the range of 0 to 100 *µ*M in a final volume of 200 *µ*L/well. After 72 h of treatment, a freshly diluted MTT solution (20 *µ*L, 1.25 mg/mL in PBS) was added to the wells, and the plates were further incubated for 4 h. Subsequently, the medium was removed, and the formazan product was dissolved in 100 *μ*L of DMSO. The number of living cells was evaluated by measuring the absorbance at 570 nm (reference 620 nm) using an absorbance reader (Spark, TECAN). The reading values were converted to the percentage of control and IC_50_ values calculated from the curves constructed by plotting the number of living cells (%) versus drug concentration (*μ*M) (IC_50_ = concentration of the compound inhibiting cell growth by 50%). Concentrations of Pt complexes present in the medium during treatment were always verified by flameless atomic absorption spectrometry (FAAS).

### 2.7. Cellular Uptake

Cellular accumulations of studied complexes were determined as already described earlier [31]. Briefly, MDA-MB-231 cells were seeded on 100 mm Petri dishes (1 × 10^6^ cells per dish). After overnight preincubation, the cells were exposed to platinum compounds for 5 or 24 h. After the treatment, the cells were detached by trypsinization, exhaustively washed with ice-cold PBS, counted using cell counter, and collected by centrifugation. Finally, the cell pellets were digested using a microwave acid (HCl) digestion system (CEM Mars). The quantity of metal taken up by the cells was determined by ICP-MS.

### 2.8. Quantification of Platinum Bound to DNA Isolated from the Cells

MDA-MB-231 cells were cultured, treated with platinum complexes, and collected as described above. The cell pellets were lysed in DNAzol (DNAzol, MRC) supplemented with RNase A (100 *µ*gmL^−1^). Genomic DNA was precipitated from the lysate by ethanol (100%), washed twice with 75% ethanol, and resuspended in 8 mM NaOH. The DNA content in each sample was determined by UV spectrophotometry. To avoid the interference of high DNA concentration on the detection of platinum in the samples, the DNA samples were digested in the presence of 30% hydrochloric acid (Suprapur, Merck Millipore). The amount of metal bound to nucleic acids was quantified by ICP-MS.

### 2.9. Measurement of the Partition Coefficients

The partition coefficients (P) of the investigated platinum complexes were determined by the “shake-flask” method. The complexes were dissolved in octanol-saturated water (OSW) containing either 10 mM KI (compounds **1a**,**b**) or 10 mM KCl (compounds **2a,b**) and mixed with water-saturated octanol (WSO). After 30 min of mixing at room temperature, centrifugation was done at 3000 g for 5 min to separate the two phases. The layers were separated carefully using a fine-tip pipette, and the water fraction was analyzed for metal content by flameless atomic absorption spectroscopy (FAAS). The partition coefficients were calculated as the ratio of the concentration of the compound in the octanol layer to that in the aqueous layer: log *P* = log([Pt]_WSO_/[Pt]_OSW_) = log(([Pt]_init_ – [Pt]_osw_)/[Pt]_OSW_).

### 2.10. DNA Binding in a Cell-Free Medium

Calf thymus DNA (64 *μ*gmL^−1^) was incubated with platinum complexes at their 20 *µ*M concentration in 10 mM NaClO_4_ at 37°C for 24 h. After that, the free (unbound) platinum in these samples was removed by gel filtration using Sephadex G-25 columns, and the samples were redissolved in double-distilled water. UV and flameless atomic absorption spectrophotometry were used to determine DNA concentration and platinum contents in these samples, respectively.

### 2.11. Reaction with GSH

The interaction of platinum complexes to GSH was followed by recording UV absorption, as already described [32, 33]. The absorbance at 260 nm was monitored as a function of time using a Beckman 7400 DU spectrophotometer equipped with a thermoelectrically controlled cell holder. Reactions were carried out in the dark at 37°C. The experimental procedure was as already outlined by Dabrowiak et al. [34]. To establish the rate of the initial reaction, the curve was fitted by nonlinear regression to the equation Id = C + A_1_exp(−b1t) + A2exp(−b2t), where A1, A2, b1, b2; C are constants, and *t* is the time of the reaction, by using GraphPad Prism software.

### 2.12. Real-Time Cell Growth Monitoring

For monitoring the growth of MDA-MB-231 cells in real-time (RTCA), a realtime cell analyzer (xCELLigence RTCA SP Instrument, ROCHE) was employed. After the background of E-plate reading (100 *μ*L medium), the cells were added to E-plate (3 × 10^3^ cells/well; 50 *μ*L) and grown for 22−24 h. Then, the investigated compounds at various concentrations were added to 50 *μ*L of media. The impedance was monitored for additional 4 days. An arbitrary unit CI (cell index) is a quantitative measure reflecting the status of the cells (number of attached cells and cell status such as morphology) in an electrode-containing well. Normalized CI at a certain time point is calculated as CI at the time point divided by CI at a reference time point.

### 2.13. Analysis of the Cell Cycle

MDA-MB-231 cells were seeded at a density of 2.5 × 10^5^ cells/dish in 35 mm Petri dishes and preincubated overnight. The cells were then treated with the investigated compounds for 24 or 48 h. Treated and untreated (control) cells were harvested by trypsinization, washed twice with PBS, resuspended in 70% ethanol, and kept at 4°C overnight. Fixed cells washed twice in PBS were stained with propidium iodide (50 *μ*gmL^−1^) in Vindel's solution (10 mM Tris-Cl, pH 8.0, 10 mM NaCl, 0.1% Triton X-100, and 100 *μ*g mL^−1^ RNase A) for 30 min at 37°C. Cell cycle profiles were measured with a FACS Verse flow cytometer (Becton Dickinson, Germany) and data analyzed using FCS Express (De Novo Software, CA).

### 2.14. Imaging of Spindle Organization

MDA-MB-231 cells (1.5 × 10^5^ cells) were seeded on glass coverslips (Marienfeld Superior cover glasses 22 × 22 mm, thickness No1, Paul Marienfeld GmbH & Co. KG, Germany) coated with 0.1% gelatin from bovine skin Type B (Sigma, Prague) placed in 6-well culture dishes and grown overnight. The cells were then treated with the tested compounds for 24 h. Following the treatment, the cells were washed and fixed with p-formaldehyde (4% in PBS; 10 min) and permeabilized with 0.1% Triton X-100 for 20 min. Following the permeabilization, the blocking solution was applied (5% goat serum in PBST) for 1 h. As a next step, the primary antibody (anti-*β* I Tubulin antibody Abcam, dilution 1 : 500) was added and incubated at room temperature for 2 h. After four washes with PBST, the secondary antibody was applied (Goat Anti-Rabit-Alexa Fluor 488, Abcam, dilution 1 : 200) and incubated for 1 h. After exhaustive washing (at least five times) with PBST, the cells were mounted with ProLong^TM^ Diamond Antifade with DAPI (4′,6-diamidino-2-phenylindole dihydrochloride, Invitrogen, Thermo Fisher Scientific, Prague, Czech Republic). The images were taken with the laser scanning confocal microscopy Leica SP-8 (Leica Microsystems GmbH, Wetzlar, Germany). DAPI was excited by blue laser (405 nm) and Alexa Fluor 488 by WLL (488 nm). Sequential detection at adequate channels (450–470 nm and 540–560 nm, respectively) was employed.

## 3. Results and Discussion

### 3.1. Synthesis and Chemical Characterization

**Figure 1:**
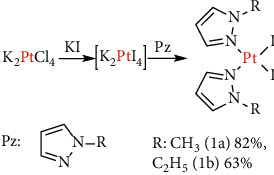
Synthesis of platinum(II) diiodido complexes.

**Figure 2:**
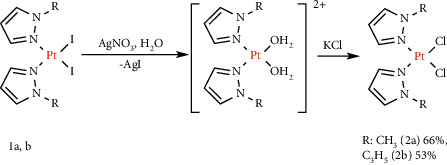
Synthesis of platinum (II) dichlorido complexes.

The ligands mepz and etpz were prepared by alkylation of the commercially available unsubstituted 1*H*-pyrazole. The platinum(II) diiodido complexes **1a**,**b** (Figure 1) were prepared using methods reported by Reedijk with a minor modification [35]. Dichlorido complexes **2a,b** (Figure 2) were prepared following Dhara's method [17]. 

The structure of all complexes was characterized by ^1^H, ^13^C, and ^195^Pt NMR spectroscopy and purity by HRMS. In ^1^H NMR spectra of **1b**, **2a,** and **2b,** we observed three signals of pyrazole ring (C^3^H, C^4^H, C^5^H). Interestingly, we found only two indistinguishable multiples from the pyrazole ring for **1a** in both DMF-*d*_7_ and acetone-*d*_6_. Coordination to the platinum center resulted in a lower field shift for pyrazole protons compared to the free ligands.

In ^195^Pt NMR spectra, we found resonance at *δ* ca. −2000 ppm for the chlorido complexes and for iodides at ca. −3200 ppm, which confirmed the complexes' cis-geometry. In the HR-ESI+ mass spectra for complexes **1a**,**b** and **2a**,**b** (Figures S1A−S1D), the most abounded peak was identified as [M + Na]^+^ or [M + H]^+^; for the **1a,b** and **2a,b**, the peak identified as [M − Hal]^+^ was also present in the spectra.

### 3.2. Stability

The stability of complexes **1a,b,** and **2a,b** in the presence of water was examined by ^1^H NMR (40% DMF-*d*_7_/60% D_2_O). We found that the chlorido complexes **2a,b** are stable in the used water-containing solvent mixture because no new signals were shown in the ^1^H NMR spectra (Figure S2). The characteristic signal of the C^4^–H pyrazole ring was detected at 6.44 ppm (for **2a**) and 6.47 ppm (for **2b**). In contrast, the new ^1^H NMR signals were detected in the spectra of both iodido complexes **1a,b** under the aqueous conditions. For example, the characteristic C^4^–H signal, which was detected at 6.45 ppm and 6.49 ppm for the parent complexes **1a** and **1b**, respectively, was also observed at 6.30 ppm in the spectra of both complexes (Figure S2). This signal (6.30 ppm) is attributable to the released pyrazole derivatives mepz (for **1a**) and etpz (for **1b**) because the signal positions correlate well with those of free pyrazoles (studied for comparative purposes). For **1a**, *ca.* 15% and 30% (*t* = 5 and 24 h, respectively) of the initial complex released its N-donor ligand mepz, while complex **1b** is somehow less stable, *ca.* 28 and 42% (*t* = 5 and 24 h, respectively) of the free ligand is released.

Although the nature of the processes connected with the ligand release is not the same for cisplatin (i.e., Pt–Cl bond hydrolysis) and **1a,b** (N-donor ligand release), both lead to the activated species capable of interacting with various biomolecules. However, the kinetics of these processes seems to be different. In particular, the cisplatin hydrolysis with *t*_1/2_ ≈ 2 h [36–38] is a much faster process than the release of pyrazole derivatives mepz and etpz from complexes **1a,b** (*t*_1/2_ > 24 h). It is worth noting that a release of N-donor ligand was recently reported for Pt(II) diiodido complexes of different structural types, specifically for *trans*-diiodidoplatinum complexes [21, 39] and unsymmetrical *cis*-ammine-diiodidoplatinum [40] complexes.

In general, chlorido derivatives of cisplatin are usually less hydrolytically stable than their iodido analogs [20, 41], which contradicts the results describing the stability of cis-Pt(II) alkylpyrazole complexes investigated in this work (Figure S2). Additionally, a mildly acidic environment (close to the situation in cancer cells) was shown to destabilize the ammonia ligands in *cis*-[PtI_2_(NH_3_)_2_] in contrast to cisplatin and thus favors ammonia release over iodide release [20].

### 3.3. Antiproliferative Activity

The activity of the four complexes with alkylpyrazole ligands (**1a,b,** and **2a,b**) was determined against human ovarian carcinoma cell lines A2780 and A2780cisR (sensitive and cisplatin-resistant), human breast adenocarcinoma cells MDA-MB-231, human colon carcinoma cell HCT116, and human rhabdomyosarcoma RD cells, commonly used to test the cytotoxic activity of cisplatin derivatives and other antitumor metallodrugs. In addition, human lung fibroblasts MRC5 pd30 were also included in the analysis as a model of noncancerous, healthy cells. The cell lines were incubated with platinum complexes for 72 h, and the number of viable cells in the cultures was evaluated by MTT assay as described in the experimental section (Materials and Methods). The resulting IC_50_ values are summarized in Table 1.

The data revealed that both iodido complexes **1a** and **1b** were significantly more effective than their chlorido counterparts **2a** and **2b**. Importantly, they were also significantly more effective than clinically used cisplatin against all inherently cisplatin-resistant cell lines tested. In contrast, chlorido derivatives showed activity comparable to cisplatin in most of the cells tested (with one exception in cisplatin-sensitive A2780 cells). Notably, all alkylpyrazole complexes were effective in overcoming acquired cisplatin resistance; iodido derivatives **1a** and **1b** are even more effective compared to chlorido complexes **2a,b**. This suggests that the mechanism underlying the biological action of the new alkylpyrazole Pt(II) complexes is likely to be somewhat different from that of cisplatin, allowing the compounds to successfully overcome the resistance mechanisms that work with cisplatin. Notably, the antiproliferative activity of Pt(II) alkylpyrazole complexes was considerably higher in tumor cells compared to nontumorigenic normal MRC5 pd30 cells (Table 1).

### 3.4. Intracellular Accumulation of Platinum

An essential step for the biological effect of platinum drugs is the efficient uptake of these drugs through the plasma membrane and the subsequent accumulation in cells. Therefore, the intracellular platinum concentration in the cells treated with the investigated complexes was determined to estimate the possible impact of the cellular uptake of these compounds on their efficacy against cancer cells.

Cell platinum levels were measured after 5 h and 24 h of exposure of MDA-MB-231 to **1a, 1b, 2a, 2b** and cisplatin at their equimolar (10 *μ*M) concentrations by ICP-MS analysis. It was verified that the cell viability was at least 85% under the conditions of these experiments, as measured by the trypan blue exclusion assay. Thus, total cell uptake was not significantly affected by the dying/dead cell membrane permeability impairment.

**Figure 3:**
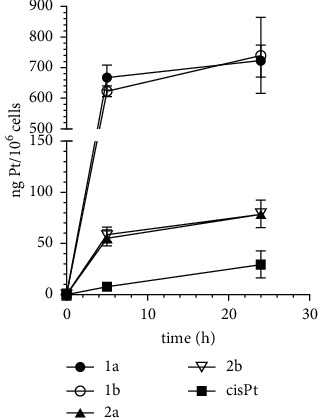
Cellular accumulation of Pt in MDA-MB-231 cells treated for 5 h and 24 h with the investigated Pt complexes.

The results expressed as ng Pt per 10^6^ cells are shown in Figure 3 and Table S1. Consistent with the antiproliferative potency, the cellular Pt content increased in the order of cisplatin < **2a** ∼ **2b** < **1a** ∼ **1b** and correlated with their lipophilicity (Table S1). The most lipophilic complex contains the ethyl group at pyrazole ring and iodido ligands and changes to chlorido ligands, or the methyl group decreases lipophilicity. Interestingly, the results in Figure 3 show that incubating the cells for more than 5 h increased the accumulation of platinum from the complexes only relatively slightly. This result can be interpreted to mean that the incubation of the cells for 5 h was sufficient for almost total platinum accumulation.

However, the correlation between cytotoxic efficacy and intracellular Pt content is not straightforward. The data presented in Table S1 and Figure 3 also show that the accumulation of Pt from complexes **2a,b** in MDA-MB-231 cells significantly exceeded the accumulation from cisplatin, although the antiproliferative activity of the three compounds was approximately the same (Table 1). Similarly, complexes **1a,b** were about 5-fold more active in MDA-MB-231 cells than cisplatin, although the intracellular level of Pt from **1a,b** was about 90-fold higher. Thus, these estimates suggest that significantly higher levels of Pt(II) alkylpyrazole complexes are required to achieve a certain level of biological activity compared to cisplatin. Furthermore, this suggests that the mechanism underlying the activity of Pt(II) alkylpyrazole complexes differs from the mechanism of the antitumor effect of cisplatin.

### 3.5. DNA Interactions

Nuclear DNA has been shown to be a major pharmacological target of cisplatin, so the anticancer activity of conventional platinum anticancer drugs is attributed to their binding to DNA [1, 3]. As Pt(II) complexes with alkylpyrazole ligands can be considered cisplatin analogs, we hypothesized that DNA could also play an important role in the activity of these compounds. Therefore, further experiments were focused on DNA binding in the cell-free medium and subsequently in the cellular environment.

**Figure 4:**
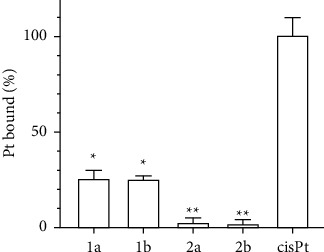
DNA binding of complexes **1a**, **1b**, **2a**, **2b**, and cisplatin with double-helical calf thymus DNA (c(Pt)/c(DNA nucleotides) = 0.02) in 10 mM NaClO_4_ at 37°C. Stars at the top of the bars indicate a statistically significant difference from cisplatin (^*∗*^*p* ≤ 0.05, ^*∗∗*^*p* ≤ 0.01). Data represent a mean ± SEM of two independent experiments, each measured in triplicate.

We first focused on quantifying the binding of cis-Pt(II) alkylpyrazole complexes to mammalian (calf thymus) double-stranded DNA in a cell-free medium, as described in Materials and Methods. The amount of platinum bound to DNA after 24 h of incubation in 10 mM NaClO_4_ is plotted in Figure 4. As expected, cisplatin binds DNA quantitatively after 24 h, consistent with previously published data [42]. However, in contrast to cisplatin, the DNA binding of all Pt(II) alkylpyrazole complexes was much less efficient. In particular, chloride derivatives **2a** and **2b** bound DNA poorly so that the level of platinum associated with DNA was almost below the detection limit of FAAS. Iodide analogs **1a** and **1b** were able to bind DNA under experimental conditions but to a much lesser extent than cisplatin. These results correlate and can be related to the above observation that chlorido complexes **2a**,**b** do not hydrolyze at all under the aqueous conditions, and the N-donor ligand (i.e., mepz and etpz) release from the iodido complexes is slower than the hydrolysis reported for cisplatin (Figure S2).

Thus, this experiment revealed that the ability of Pt(II) alkypyrazole complexes to bind to DNA in a cell-free medium is significantly reduced compared to cisplatin. However, the situation in living cells may differ from extracellular conditions, and hydrolytic processes may vary significantly due to the more complex intracellular environment. Therefore, DNA platination in living cells was also determined.

**Figure 5:**
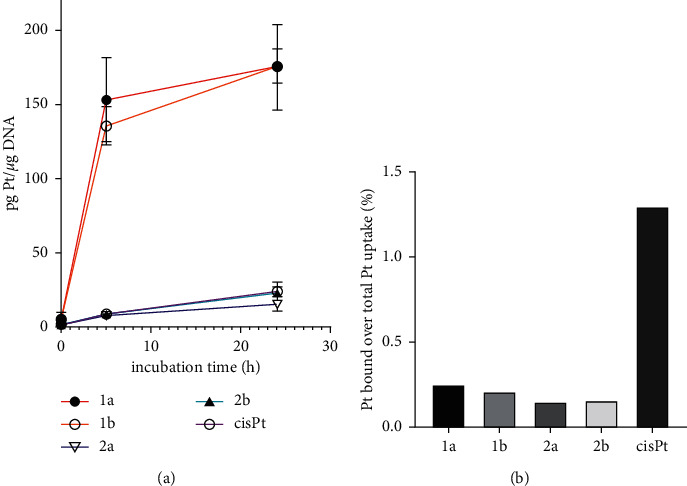
(a) DNA platination in MDA-MB-231 cells treated for 5 and 24 h at 10 *µ*M concentrations of Pt(II) complexes. All results are expressed as the mean ± SD from three independent experiments. (b) The percentage of Pt bound to DNA over the total Pt taken up to MDA-MB-231 cells (5 h incubation).

As shown in Figure 5(a) and Table S2, the level of platinum associated with nuclear DNA in the treated cells was significantly higher for the iodido complexes **1a** and **1b** than in the chlorido analogs **2a** and **2b**, as well as for cisplatin. Thus, the trend in intracellular DNA platination of the investigated complexes roughly follows their cellular uptake and accumulation. However, evaluation of the efficiency of intracellular DNA platination (the amount of platinum bound to DNA versus the total amount of platinum in the cells) showed remarkable results (Figure 5(b)). Of the total Pt inside MDA-MB-231 cells, a significantly higher fraction of platinum from cisplatin was bound to DNA than **1a**,**b,** and **2a**,**b**. These fractions represent 1.3% (cisplatin), 0.25% (**1a**), 0.21% (**1b**), 0.15% (**2a**), and 0.16% (**2b**) of the total Pt accumulated in cells. In aggregate, the yield of platination of DNA by Pt(II) alkylpyrazole complexes was significantly (5-9-fold) lower than that of cisplatin. These results correlate with a reduced ability of new Pt-alkylpyrazole complexes to bind DNA found in a cell-free medium.

### 3.6. Influence of DNA Nucleotide Excision Repair on Biological Activity

The results demonstrating DNA platination by the investigated Pt-alkylpyrazole complexes in the cell-free medium or cells (Figures 4 and 5) suggest that, in contrast to antitumor effects of cisplatin, DNA may not be such an important target for the Pt(II) complexes investigated in this study. A generally accepted criterion that proves that DNA is the target is based on the observation that the investigated drug exhibits higher toxicity in cells that are deficient in DNA repair [1] because the persistence and toxicity of DNA lesions depend on the ability of the cells to repair the damage. Therefore, the pair of Chinese hamster ovary cells CHO-K1 line (wild-type) and its isogenic mutant line MMC-2 (nucleotide excision repair (NER) deficient, carrying the ERCC3/XPB mutation) was used to distinguish whether DNA damage is involved in the mechanism of action of the new Pt(II) alkylpyrazole complexes.

The data in Table 2 show that DNA repair-deficient MMC-2 cells were significantly more sensitive (11-fold) to cisplatin than wild-type CHO-K1 cells, consistent with previously published results [30]. However, when the cells were treated with Pt(II) alkylpyrazole complexes investigated in this work, the difference in the sensitivity of repair-deficient cells and repair-proficient cells decreased markedly, suggesting that unrepaired DNA lesions formed by the investigated Pt-alkylpyrazole complexes contribute markedly less to their cytotoxicity than those formed by cisplatin. Thus, DNA damage is unlikely to be a decisive factor responsible for the activity of the investigated Pt(II)-alkylpyrazole complexes.

### 3.7. Interaction with GSH

Before platinum drugs reach the DNA in the nucleus of tumor cells, they can interact with various sulfur-containing molecules because Pt(II) compounds show a strong thermodynamic preference for binding to sulfur donor ligands [43]. These interactions are generally thought to play a role in the mechanisms underlying the activity (inactivation) of Pt drugs [43,44]. The study of the interactions of platinum antitumor complexes with sulfur-containing compounds of biological importance may help elucidate other aspects of the action of the new Pt compounds. So the reactions of cis-Pt(II) alkylpyrazole complexes with GSH were investigated using the methods already described [34].

**Figure 6:**
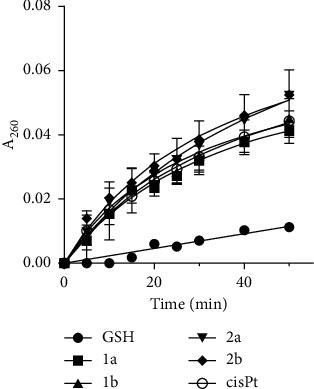
UV absorbance associated with the reaction of complexes **1a**, **1b**, **2a**, **2b**, and cisplatin with GSH. Absorbance at 260 nm is shown as a function of time. The curves represent absorbances of the solution containing platinum complex plus GSH, from which the absorbance yielded by the Pt complex and GSH at *t* = 0 was subtracted. All results are expressed as the mean ± SD from two independent experiments, each performed in triplicate.

Complexes **1a**,**b**, **2a**,**b** or cisplatin were incubated with GSH in a thiol to drug ratio of 500:1, which is a physiologically relevant value [34].  Figure 6 shows the UV absorbance (at 260 nm) of the platinum complexes and GSH as a function of time, subtracting the absorbance of the platinum complex itself. To determine the rate of initial reaction with respect to the platinum complex, each difference curve was fitted by nonlinear regression (GraphPad Prism) to the following equation: I_d_ = C + A_1_exp(−b_1_*t*) + A_2_exp(-b_2_*t*), where *A*_1_, *A*_2_, *b*_1_, *b*_2_, and C are constants and *t* is the reaction time. The initial slope (S_in_) was calculated as A1b1 + A2b2 [34]. The values of S_in_ values of (7.6 ± 0.9) × 10^−4^, (8.4 ± 0.9) × 10^−4^, (7.1 ± 0.5) × 10^−4^, (8.6 ± 0.4) × 10^−4^, and (7.9 ± 0.5) × 10^−4^ were calculated for the reaction of GSH with cisplatin, **1a**, **1b**, **2a**, and **2b**, respectively.

Remarkably, we have found that the pattern of GSH interaction of iodido complexes **1a** and **1b** was almost superimposable to that of cisplatin. The interaction rates of complexes **2a** and **2b** appear to be slightly faster, but these differences are not statistically significant (Figure 6). As the absorbance at 260 nm reflects the presence of platinum-sulfur and disulfide bonds [34,45], the observations can be interpreted to mean that under the conditions of our experiment, all four investigated Pt-alkylpyrazole complexes react with GSH at a similar initial rate. It implies that differences in the biological activity of these complexes are unlikely to be related to their different inactivation by GSH.

### 3.8. The Effect on the Organization of the Mitotic Spindle and the Cell Cycle

**Figure 7:**
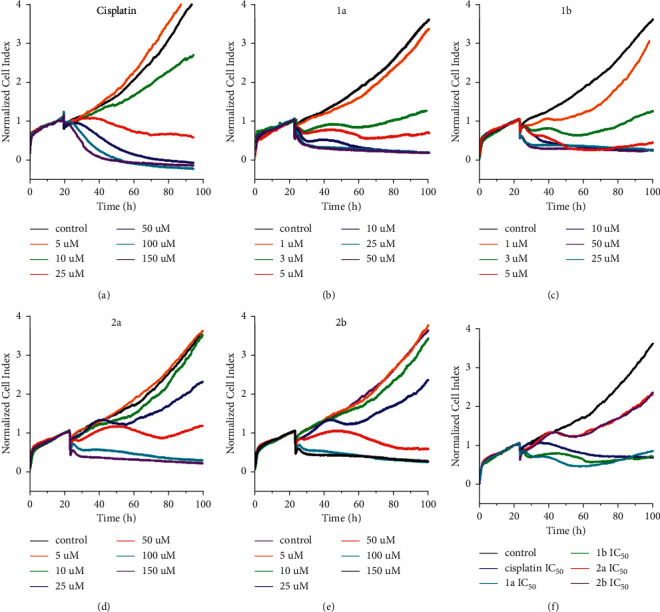
Interaction of MDA-MB-231 cells with cisplatin (a), **1a** (b), **1b** (c), **2a** (d), and **2b** (e) monitored by a real-time cell analyzer (RTCA). (f) A comparison of RTCA profiles was found for **1a**, **1b**, **2a**, **2b** and cisplatin at their equitoxic concentrations (IC_50,72h_). The vertical lines indicate the start of the treatment after allowing the cells to adhere to microelectrodes and grow for 24 h. Cell indices were normalized to account for differences in cell counts that existed across the wells before the treatment. Incubations were performed in triplicate with 3000 cells per well.

The results mentioned above revealed that DNA damage, although contributing to overall activity, is unlikely to be a predominant factor underlying the biological activity of the investigated Pt-alkylpyrazole complexes. Therefore, further steps have been taken to further elucidate the mechanism of action of the compounds. Impedance monitoring of cellular responses was used for this purpose. It has been shown [46–48] that biologically active compounds produce time-dependent cell response profiles (TCRPs), which can be predictive of the mechanism of action of the investigated molecules. The TCRPs of cisplatin and Pt-alkylpyrazole complexes **1a**, **b** and **2a**, **b** are displayed in Figure 7.

Figure 7 demonstrates concentration-dependent TCRPs obtained for the investigated platinum complexes. The TCRP obtained for cisplatin at the concentration close to the IC_50_ value was characterized by an initial slight increase in the cell index (CI) in comparison with the control, followed by a time-dependent decrease in the CI below the control level (reflecting cytotoxic responses) without any indication of recovery of CI (see the red curve in Figure 7(a) or blue curve in Figure 7(f)). Thus, the TCRP of cisplatin coclustered with the TCRPs of compounds interfering with DNA synthesis and replication, transcription, and translation [46], which are also known to induce cell cycle arrest followed by the induction of cell death.

In contrast, treatment with the investigated Pt-alkylpyrazole complexes (Figures 7(b)–7(e)) resulted in TCRPs different from those typical for DNA-damaging agents, including cisplatin. The TCRPs obtained for **1a**, **1b**, **2a**, and **2b** also at the concentrations close to their IC_50_ (at equitoxic concentrations corresponding to their IC_50_ values) consisted of a time-dependent decrease in CI reaching a minimum point within ∼60–80 h after treatment and followed by recovery of the CI (see red and green curves in Figures 7(b) and 7(c) for 3 and 5 *µ*M **1a** and **1b** or blue and red curves in Figures 7(d) and 7(e) for 25 and 50 *µ*M **2a** and 2**b** or corresponding curves in Figure 7(f)). Interestingly, these profiles can be coclustered with those displayed by nontubulin-targeting mitotic inhibitors, such as monastrol and S-trityl-cysteine, which inhibit the mitotic kinesin Eg5 [46]. TCRPs obtained for all investigated complexes at considerably higher concentrations (Figures 7(a)–7(e) caused the complete killing of adherent cells at the longest times of cell growth. The finding that TCRPs obtained for Pt-alkylpyrazole complexes can be coclustered with those displayed by nontubulin-targeting mitotic inhibitors which inhibit the mitotic kinesin Eg5 was surprising, as no antitumor Pt complexes have been reported to act via this mechanism. However, a search in the literature has yielded several references suggesting that pyrazoles and their derivatives, such as dihydropyrazole, are widely recognized as potent inhibitors of Eg5 [49–52]. Thus, the TRCPs resembling Eg5 inhibition could potentially result from the activity of alkylpyrazole ligands or their metabolic products. Further experiments were therefore performed to confirm or refute this hypothesis.

Kinesin Eg5 is an essential spindle motor protein. Its important role is to assemble and maintain the bipolar spindle during mitosis, making it an attractive therapeutic target that could promote tumor growth regression [53–57]. Kinesin Eg5 inhibition stops the migration of centrosome to the polar region resulting in the formation of monoastral spindle [58, 59]. This atypical phenotype plays an essential role in activating the mitotic spindle assembly checkpoint, resulting in mitotic arrest [53, 58, 60].

**Figure 8:**
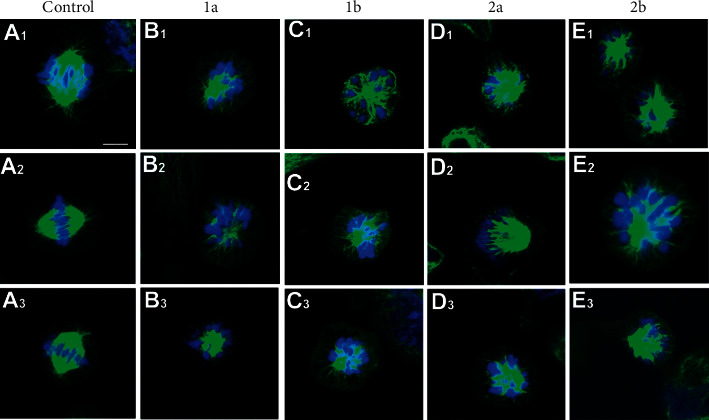
Spindle morphology of MDA-MB-231 cells untreated (control, (a)) or treated for 24 h with equitoxic concentrations (IC_50,72h_) of **1a** ((b)), **1b** ((c)), **2a** ((d)), or **2b** ((e)). Three representative images of mitotic MDA-MB-231 cells are shown, immunostained for tubulin (green) and DNA (blue); scale bar, 6 µm For better resolution, see Figure S4.

The following experiments, therefore, focused on evaluating the effect of the investigated Pt-alkylpyrazole complexes on the organization of the mitotic spindle and the cell cycle.  Figure 8 shows representative images of mitotic MDA-MB-231 cells immunostained for *β*-tubulin (green) and DNA (blue). As indicated, control untreated cells form uniformly bipolar spindles of normal morphology (Figure 8(a)). However, complexes **1a**, **b** and **2a**, **b** used at equitoxic concentrations caused monopolar (monoastral) spindles formation in MDA-MB-231 (Figures 8(b)–8(e)), similar to monastrol and other EG5 inhibitors. This effect is consistent with weak-binding motors being unable to resist inward-directed spindle forces [61]. Interestingly, all mitotic cells in the investigated samples showed solely monoastral (symmetrical or asymmetrical) spindles. These data validate our results obtained from the TCRPs assay, indicating that these compounds are indeed effective inhibitors of Eg5 activity.

**Figure 9:**
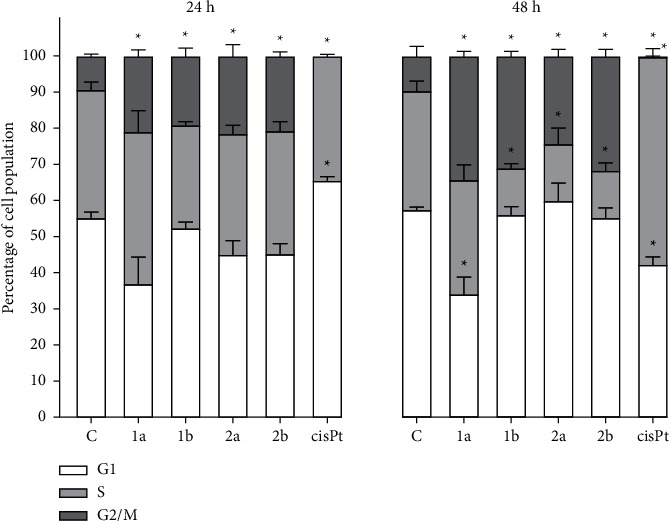
Effect of Pt-alkylpyrazole complexes on cell cycle distribution: quantitative analysis of cell cycle phases proportion. MDA-MB-231 cells were either untreated or treated with equitoxic concentrations (IC_50,72h_) of investigated Pt complexes or cisplatin for 24 h or 48 h. Data represent the mean (±SD) of three independent experiments; stars indicate significant difference from control, untreated cells (*p* < 0.01).

To evaluate whether Pt-alkylpyrazole compounds can disrupt normal cell cycle progression, the effect of the compounds on MDA-MB-231 cells was analyzed by flow cytometry. The results showed (Figures 9 and S3) that after treatment, all compounds **1a**, **b** and **2a**, **b** caused the cell cycle arrest at the G2/M phase, consistent with their mitotic inhibitory action predicted by TCRPs. This effect was both concentration- and time-dependent (Figures 9 and S3). In contrast, cisplatin arrests the MDA-MB-231 cell cycle in the way that cells cannot proceed to the G2/M phase according to its DNA-damaging mode of action and in accordance with already published data [62].

## 4. Conclusions

Four new analogs of conventional cisplatin have been prepared and characterized by physicochemical methods, namely, cis-Pt(II) complexes containing either methyl or ethyl pyrazole N-donor ligands and chlorido or iodido ligands. While complexes with chlorido ligands are stable for 48 h in the aqueous medium, complexes with iodido ligands undergo hydrolysis in the aqueous medium, releasing the alkylpyrazole ligand(s).

The investigated Pt(II) complexes with alkylpyrazole ligands show interesting antiproliferative activity in tumor cells (Table 1). Chlorido complexes display activity comparable to cisplatin. In contrast, iodido complexes are considerably more potent, which is most likely related to their enhanced transport into cells and cellular accumulation (Figure 3) due to their greater lipophilicity (Table S1). However, all of the studied Pt(II) alkylpyrazole complexes are less active in nontumor healthy cells than the clinically used cisplatin, thus displaying a higher selectivity for tumor cells. Also importantly, they very effectively overcome the acquired resistance to cisplatin. These initial results allowed us to suggest that the investigated Pt(II) alkylpyrazole complexes affect tumor cells by a mechanism different from that of cisplatin. This also implies that the resistance pathways that the tumor cells have developed against cisplatin were less effective if the tumor cells were treated with the investigated Pt(II) alkylpyrazole complexes.

The studied Pt(II) alkylpyrazole complexes were unable to bind DNA in cell-free media either at all (chlorido derivatives) or bound only very little (iodido derivatives). However, a considerable DNA binding of platinum was observed in the cells treated with both iodido and chlorido complexes (Figure 5(a)), probably due to the action of the cellular environment (containing enzymes and other bioactive molecules which may interact with the complexes accumulated inside cells) and consequently may activate these complexes. However, the frequency of DNA adducts formed by Pt(II) alkylpyrazole complexes in MDA-MB-231 was significantly lower than that formed by cisplatin (Figure 5(b)). In addition, a comparison of the amount of Pt per DNA with the antiproliferative activity of the platinum complexes showed that the adducts formed by alkylpyrazole complexes are much less effective in inhibiting the viability of MDA-MB-231 cells than those formed by cisplatin; much fewer DNA adducts of cisplatin were sufficient to elicit the same biological effect as DNA adducts of Pt(II) alkylpyrazole complexes. These observations, along with the markedly smaller cytotoxic contribution of the unrepaired DNA lesions formed by the investigated Pt(II)-alkylpyrazole complexes (Table 2), implicate that DNA damage by the investigated Pt(II) alkylpyrazole complexes does not play a major role in their mechanism of action, although it may contribute to a small extent.

In contrast, our results (Figure 8) have clearly shown, for the first time, that inhibition of the tubulin kinesin Eg5, which is essential for the formation of a functional mitotic spindle, plays an important role in the mechanism of antiproliferative action of platinum-based antitumor drugs. As a result, tumor cell division is inhibited. This mechanism may also explain the greater selectivity to tumor cells found for the investigated Pt(II) alkylpyrazole complexes compared to cisplatin because healthy cells do not divide or divide significantly more slowly. Eg5 is becoming an increasingly attractive therapeutic target for the treatment of cancer by chemotherapeutics [57]. Hence, the introduction of cis-Pt(II) alkylpyrazole complexes in this work may be an incentive to search for new anticancer chemotherapeutics derived from platinum complexes with this mechanism of action.

## Figures and Tables

**Table 1:** IC_50_ values^a^ (*μ*M) obtained for **1a**, **1b**, **2a**, **2b**, and cisplatin by MTT assay after 72 h of incubation.

	A2780	A2780cisR^b^	MDA-MB-231	RD	HCT116	MRC5 pd30
1a	4 ± 1	4 ± 1 (0.9)	4.2 ± 0.6	2.6 ± 0.8	2.4 ± 0.2	9 ± 1
1b	5 ± 1	6 ± 2 (1.2)	5.0 ± 0.9	2.9 ± 0.8	3.7 ± 0.6	11 ± 3
2a	11 ± 1	21 ± 2 (1.9)	29 ± 4	8 ± 2	14 ± 2	73 ± 9
2b	11.9 ± 0.9	19 ± 2 (1.6)	25 ± 3	8 ± 2	14 ± 3	69 ± 3
cis-Pt	4.0 ± 0.4	22 ± 2 (5.6)	24 ± 6	14 ± 1	11 ± 2	24 ± 1

^a^The results represent mean values ± SD of three independent experiments, each made in duplicate. ^b^Resistance factor, defined as IC_50_(A2780cisR)/IC_50_(A2780), is given in parentheses.

**Table 2:** IC_50_ values (*μ*M) and for Pt(II) alkylpyrazole complexes and cisplatin as determined in Chinese hamster ovary CHO-K1 cell line (wild-type) and its mutant cell line MMC-2 deficient in DNA nucleotide excision repair by MTT assay after 72 h treatment.

	CHO-K1	MMC2	*r * ^b^
**1a**	7.3 ± 0.8	4.9 ± 0.7	1.5
**1b**	11 ± 2	7.1 ± 0.8	1.5
**2a**	75 ± 7	38 ± 5	2.0
**2b**	62 ± 7	37 ± 6	1.7
cis-Pt	66 ± 12	6 ± 1	11

^a^The results are expressed as mean values ± SD from three independent experiments, each performed in duplicate. ^b^*r* = a ratio defined as IC_50_(NER-efficient, CHOK-1)/IC_50_ (NER-deficient, MMC-2).

## Data Availability

All data used to support the findings of this study are included within the article.

## Supplementary Materials

Supplementary Materials Figure S1: HR-ESI+ mass spectra of complexes **1a** (a) and **2a** (b). Figure S2: the ^1^H NMR stability studies of complexes **1a** (a) and **2a** (b). Table S1: cellular accumulation of Pt and log *P* values. Table S2: platinum content of DNA isolated from MDA-MB-231 cells. Figure S3: cell cycle analysis in MDA-MB-231 cells by flow cytometry.
