# Supplementary material for: Novel cis-Pt(II) Complexes with Alkylpyrazole Ligands: Synthesis, Characterization, and Unusual Mode of Anticancer Action
Source: Bioinorg Chem Appl. 2022 Mar 2;2022:1717200. doi: 10.1155/2022/1717200 (PMC8906972; doi:10.1155/2022/1717200)
Supplement: Supplementary Materials — Figure S1: HR-ESI+ mass spectra of complexes 1a (a) and 2a (b). Figure S2: the 1H NMR stability studies of complexes 1a (a) and 2a (b). Table S1: cellular accumulation of Pt and log P values. Table S2: platinum content of DNA isolated from MDA-MB-231 cells. Figure S3: cell cycle analysis in MDA-MB-231 cells by flow cytometry. [file 1717200.f1.docx]

Supporting Information

for

**Novel cis-Pt(II) Complexes with Alkylpyrazole Ligands: Synthesis, Characterization, and Unusual Mode of Anticancer Action**

Jana Kasparkova,^1^ Hana Kostrhunova,^1^ Vojtech Novohradsky,^1^ Аlexey A. Logvinov,^2^ Viktor V. Temnov,^2^ Nataliya E. Borisova,^2^ Tatiana A. Podrugina,^2^ Lenka Markova,^1^ Pavel Starha,^1^ Alexey. A. Nazarov,^2^ and Viktor Brabec^1^*

^1^ Czech Academy of Sciences, Institute of Biophysics, Brno CZ-61265, Czech Republic.
^2^ Lomonosov Moscow State University, Faculty of Chemistry, Leninskie Gory 1/3, 119991 Moscow, Russian Federation.

**Table of Contents**

HR-ESI+ mass spectra of complexes **1a**,**b** and **2a**,**b**. (Figures S1A-D) S2

The ^1^H NMR stability studies of complexes **1a**,**b** and **2a**,**b**. (Figure S2) S4

Cellular accumulation of Pt and log P values (Table S1) S4

Platinum content of DNA isolated from MDA-MB-231 cells (Table S2) S5

Cell cycle analysis in MDA-MB-231 cells by flow cytometry (Figure S3) S5

Spindle morphology of MDA-MB-231 cells untreated or treated with **1a,b** and **2a,b** S6


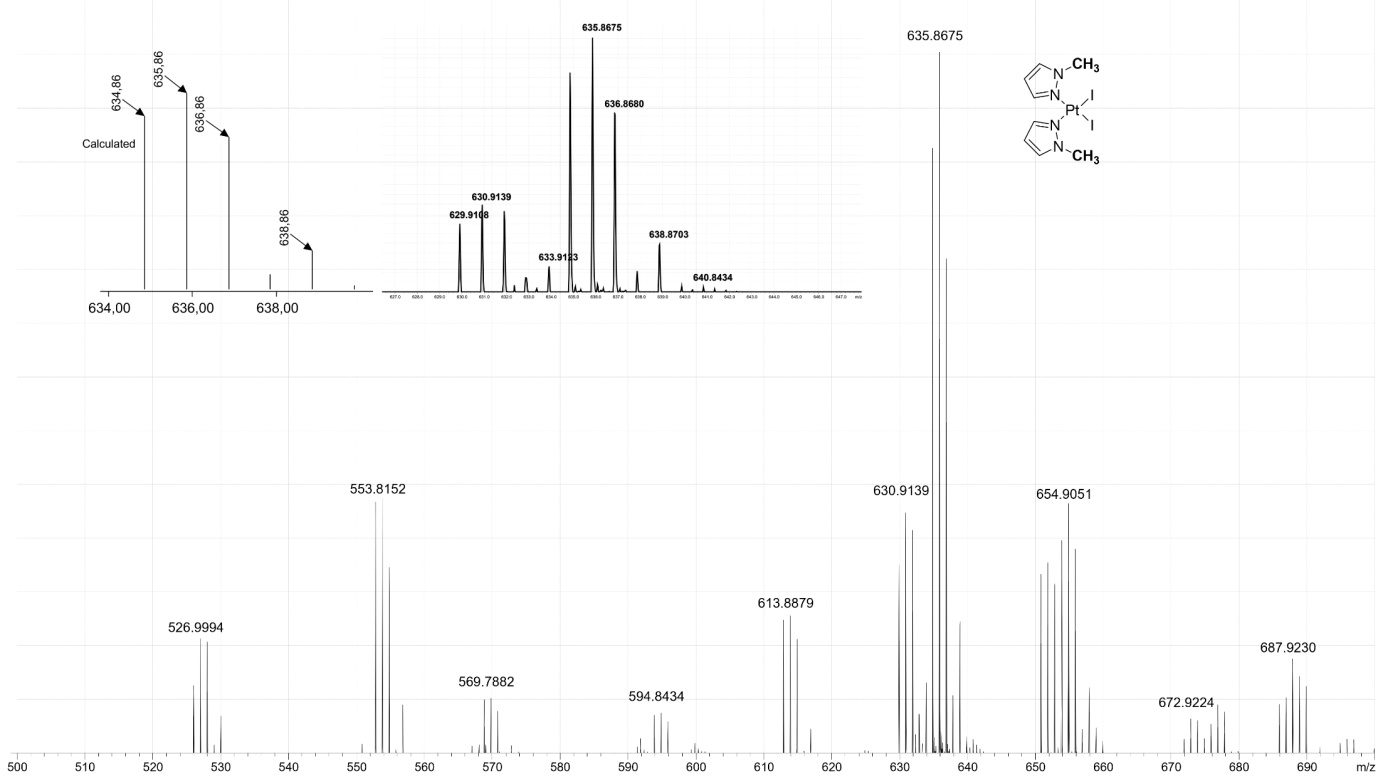


**Figure S1A:** The HR-ESI spectrum of complex 1**a** in DMF.


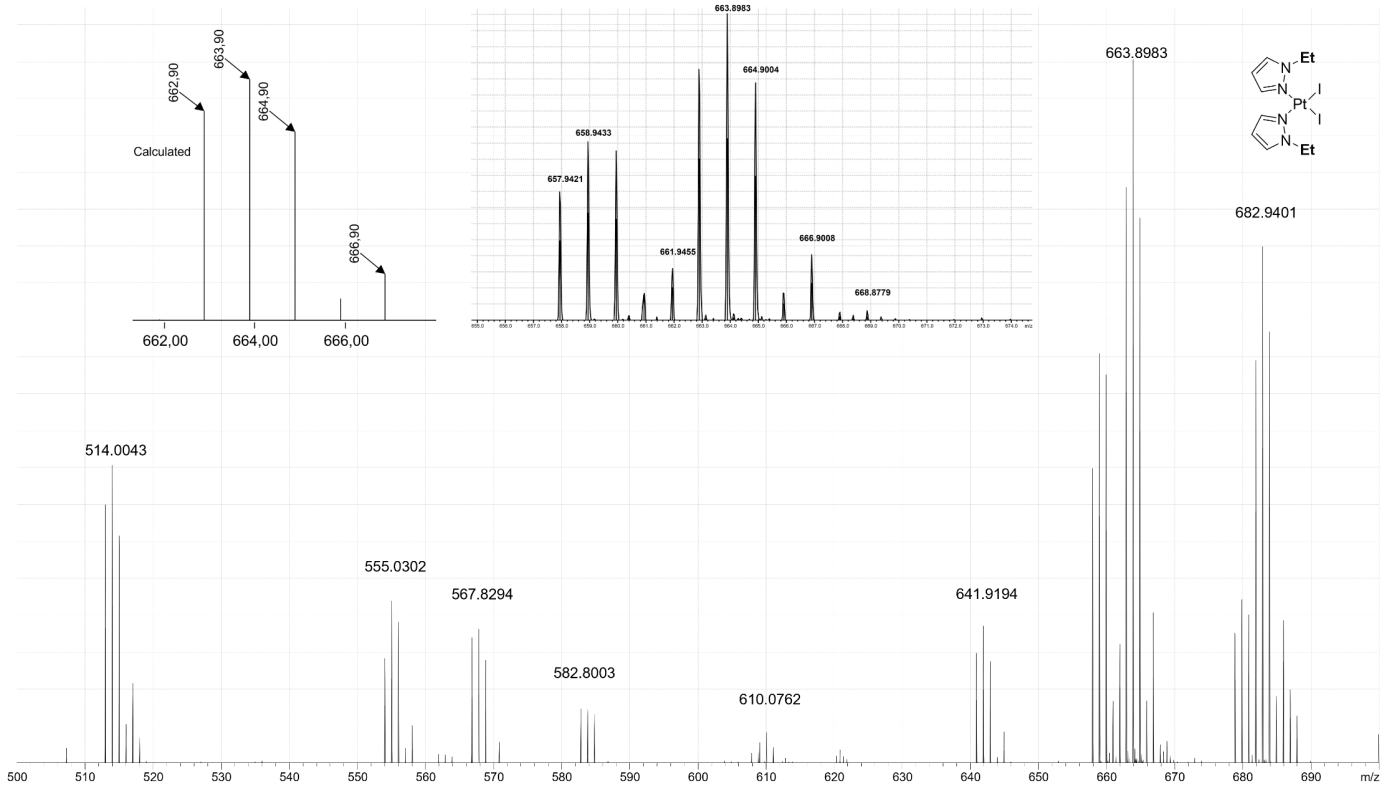


**Figure S1B:** The HR-ESI spectrum of complex **1b** in DMF**.**


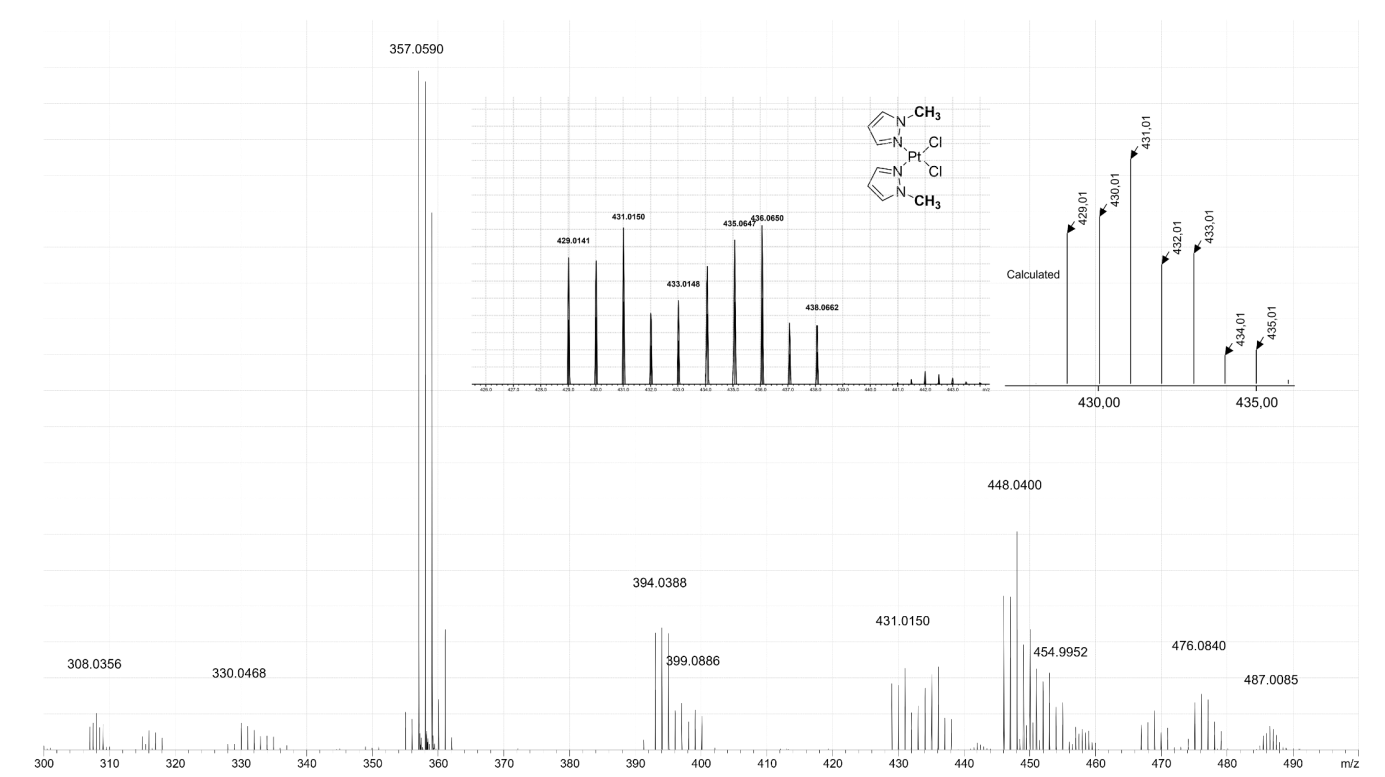


**Figure S1C:** The HR-ESI spectrum of complex **2a** in DMF.


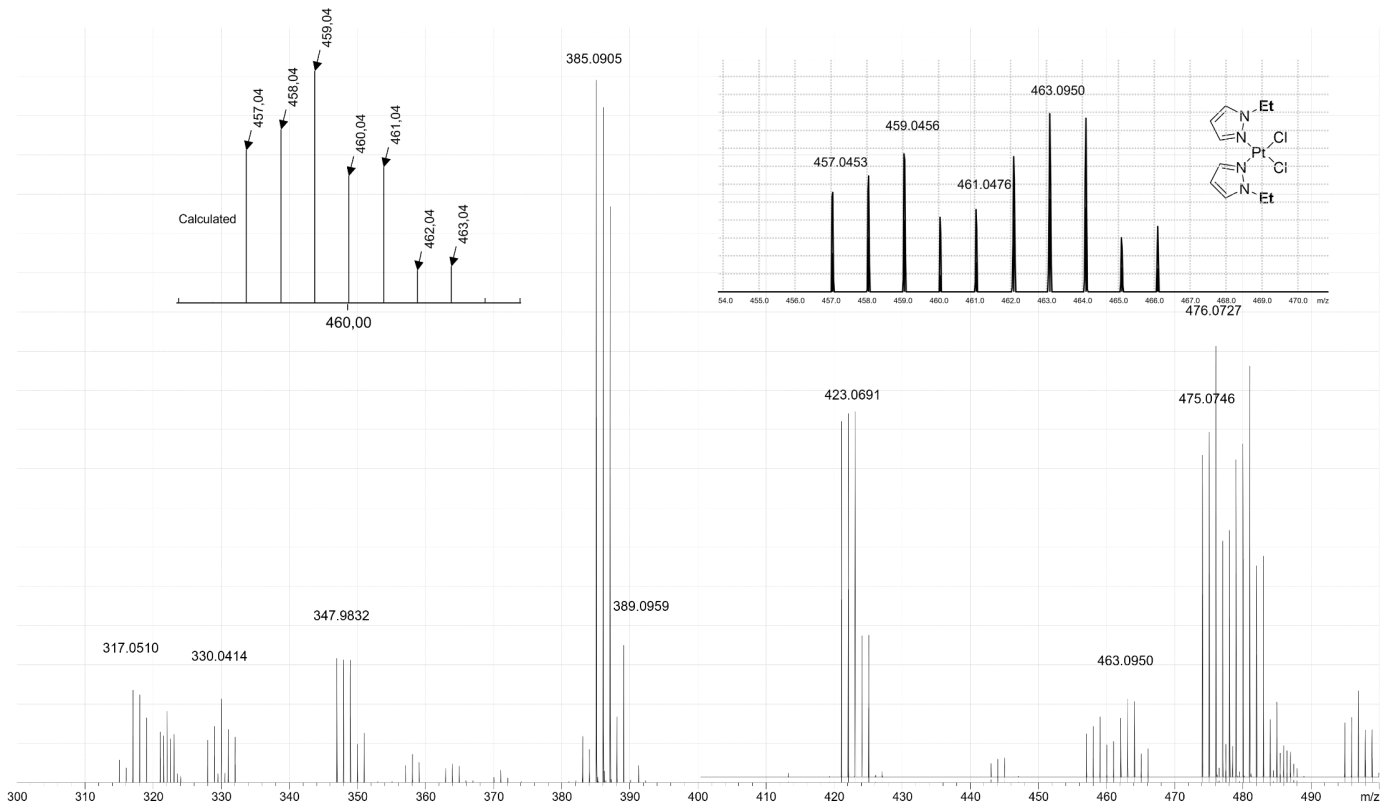


**Figure S1D:** The HR-ESI spectrum of complex **2b** in DMF**.**

**
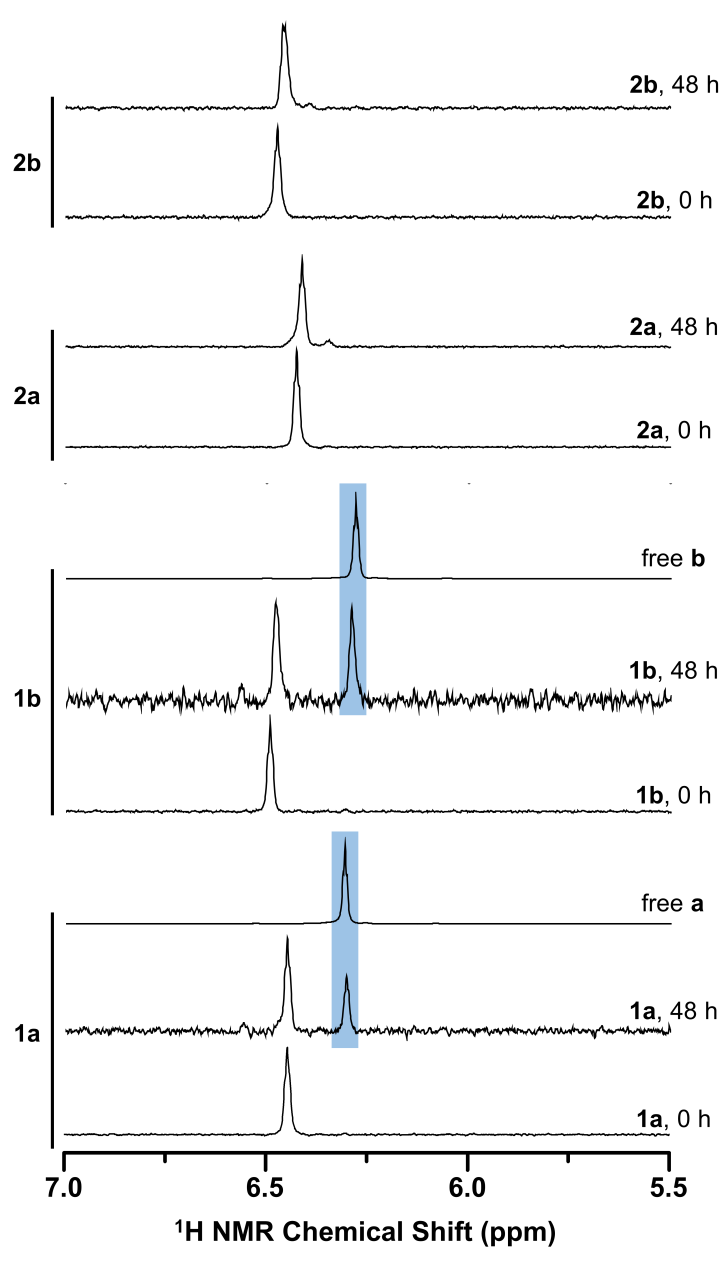
**

**Figure S2:** The ^1^H NMR stability studies of complexes **1a**,**b** and **2a**,**b** in 40% DMF-*d_7_*/60% D_2_O at different time points (0 h and 48 h). The characteristic C^4^–*H* region is depicted and the C^4^–*H* signals of free pyrazoles **a** and **b** are shown using a blue background. The spectra of free **a,b** are given for comparative purposes.

**Table S1**: Cellular accumulation of Pt (ngPt/10^6^ cells)^a^ in MDA-MB-231 cells treated for 5 or 24 h with **1a**,**b, 2a**,**b**, and cisplatin and log P octanol/water values measured by the “shake-flask” method at room temperature.

|  | **5h** | **24h** | **logP** |
| --- | --- | --- | --- |
| **1a** | 623 ± 12 | 722 ± 37 | 0.17 ± 0.08 |
| **1b** | 667 ± 29 | 739 ± 101 | 0.28 ± 0.05 |
| **2a** | 54 ± 5 | 78 ± 2 | -0.96 ± 0.05 |
| **2b** | 58 ± 5 | 78 ± 9 | -0.63 ± 0.08 |
| **cisPt** | 6.9 ± 0.2 | 19 ± 5 | -2.3^b^ |

^a^The results are expressed as the mean values ± SD from at least three measurements. ^b^Taken from [Hoeschele, 2020 #1145].

**Table S2**: Platinum content of DNA (pg Pt/µg DNA)^a^ isolated from MDA-MB-231 cells exposed to investigated Pt complexes.

|  | **5h** | **24 h** |
| --- | --- | --- |
| **1a** | 153 ± 28 | 183 ± 29 |
| **1b** | 136 ± 13 | 176 ± 16 |
| **2a** | 8 ± 2 | 16 ± 4 |
| **2b** | 9 ± 1 | 24 ± 3 |
| **cisPt** | 8.8 ± 0.6 | 24 ± 5 |

^a^The results are expressed as the mean values ± SD from at least three measurements.


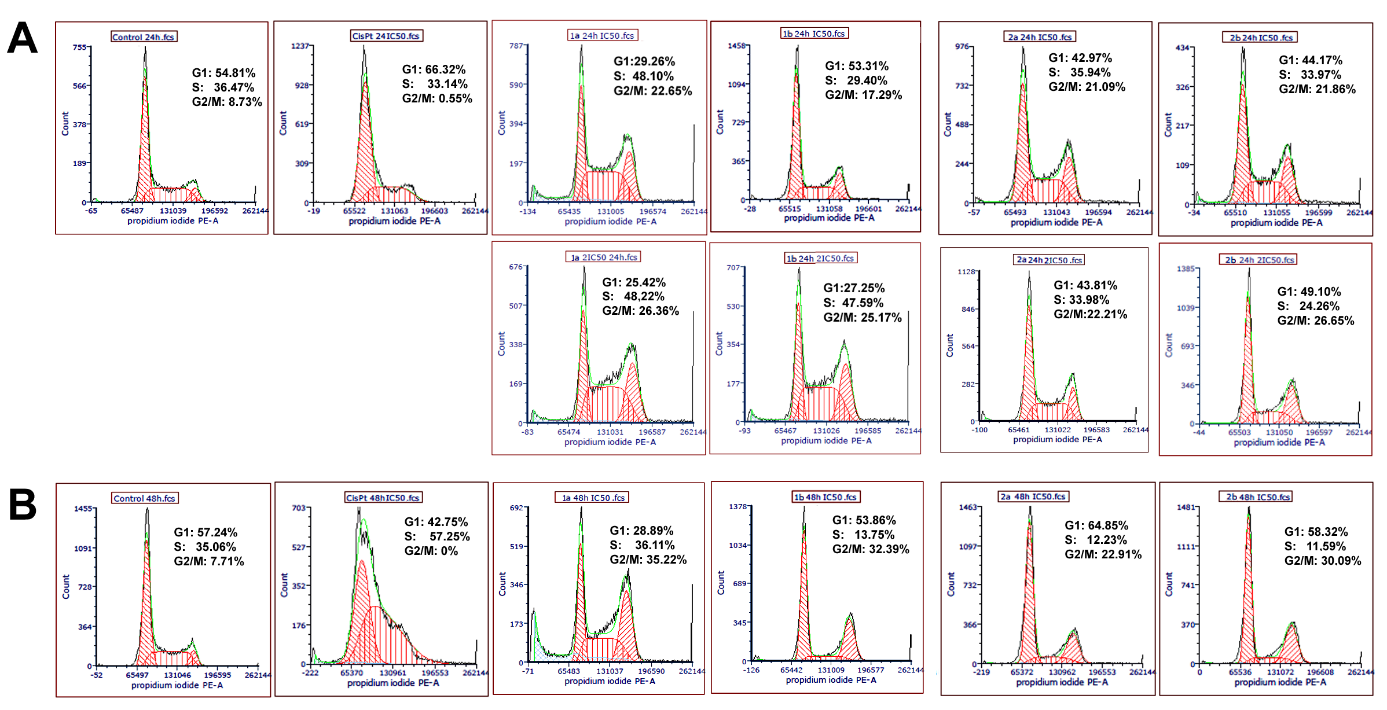


**Figure S3**: Representative cell cycle analysis performed by flow cytometry following staining with propidium iodide in MDA-MB-231 cells untreated (control) or treated with investigated complexes and cisplatin for 24h (panels A) or 48h (panels B) at their equitoxic concentrations corresponding to IC_50,72h_ or 2xIC_50,72h._

_
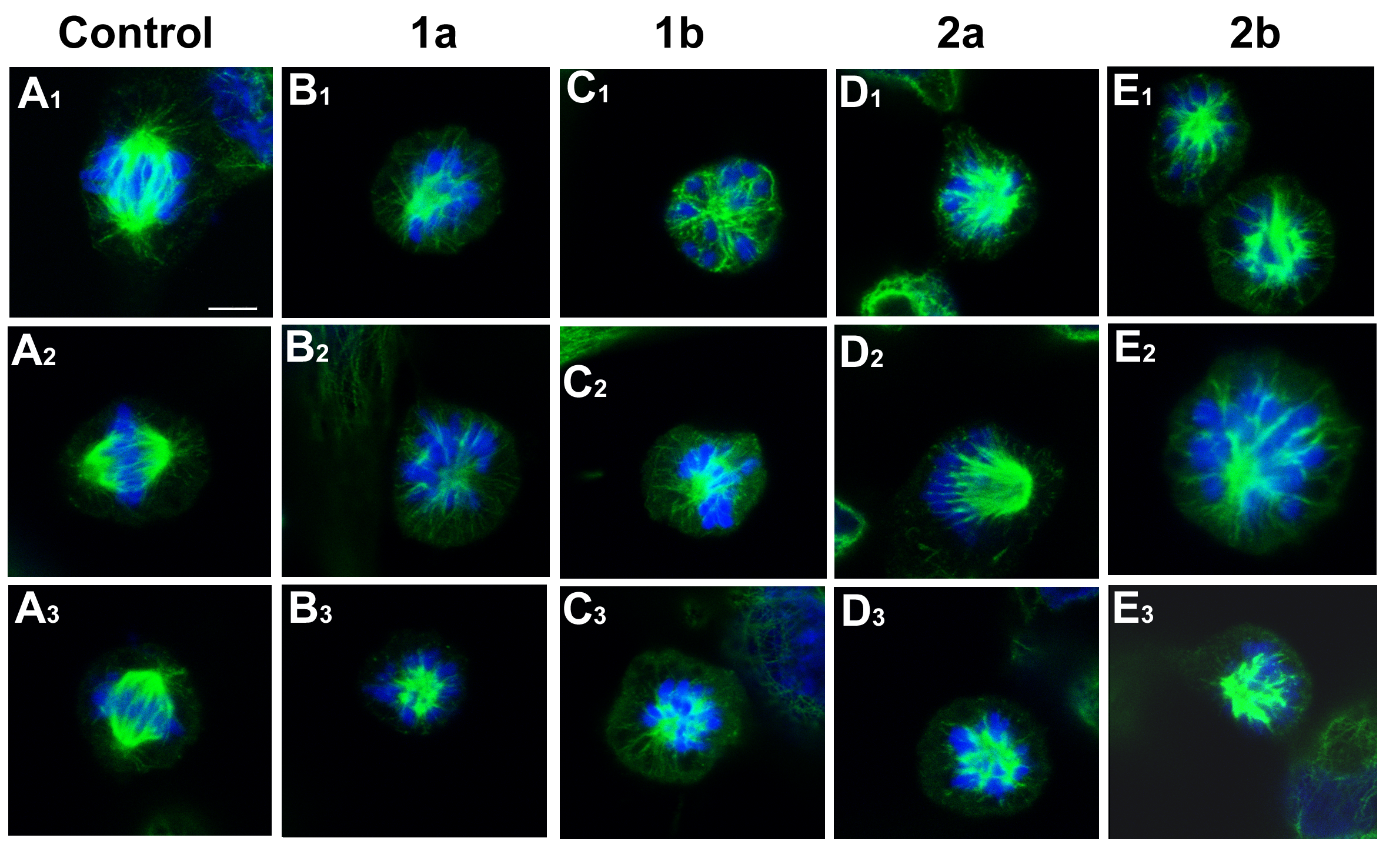
_

**Figure S4**. Spindle morphology of MDA-MB-231 cells untreated (control, panels A), or treated for 24h with equitoxic concentrations (IC50,72h) of 1a (panels B), 1b (panels C), 2a (panels D), or 2b (panels E), Three representative images of mitotic MDA-MB-231 cells are shown, immunostained for tubulin (green), and DNA (blue); scale bar 6 µm.
